# Decoding the Mechanism of Huanglian Jiedu Decoction in Treating Pneumonia Based on Network Pharmacology and Molecular Docking

**DOI:** 10.3389/fcell.2021.638366

**Published:** 2021-02-18

**Authors:** Xianhai Li, Hua Tang, Qiang Tang, Wei Chen

**Affiliations:** ^1^School of Pharmacy, Chengdu University of Traditional Chinese Medicine, Chengdu, China; ^2^Innovative Institute of Chinese Medicine and Pharmacy, Chengdu University of Traditional Chinese Medicine, Chengdu, China; ^3^School of Basic Medical Sciences, Southwest Medical University, Luzhou, China; ^4^School of Life Sciences, North China University of Science and Technology, Tangshan, China

**Keywords:** bacterial pneumonia, network pharmacology, GO, KEGG, molecular docking

## Abstract

Huang-Lian-Jie-Du decoction (HLJDD) has been used to treat pneumonia for thousands of years in China. However, our understanding of its mechanisms on treating pneumonia is still unclear. In the present work, network pharmacology was used to analyze the potential active ingredients and molecular mechanisms of HLJDD on treating pneumonia. A total of 102 active ingredients were identified from HLJDD, among which 54 were hit by the 69 targets associated with pneumonia. By performing Gene Ontology (GO) and Kyoto Encyclopedia of Genes and Genomes (KEGG) enrichment analysis, we obtained the main pathways associated with pneumonia and those associated with the mechanism of HLJDD in the treatment of pneumonia. By constructing the protein–protein interaction network of common targets, 10 hub genes were identified, which were mainly involved in the tumor necrosis factor (TNF) signaling pathway, interleukin 17 (IL-17) signaling pathway, and nucleotide-binding oligomerization domain (NOD)-like receptor signaling pathway. Moreover, the results of molecular docking showed that the active ingredients of HLJDD had a good affinity with the hub genes. The final results indicate that HLJDD has a greater effect on bacterial pneumonia than on viral pneumonia. The therapeutic effect is mainly achieved by regulating the host immune inflammatory response and oxidative stress reaction, antibacterial microorganisms, alleviating the clinical symptoms of pneumonia, repairing damaged cells, and inhibiting cell migration.

## Introduction

Pneumonia refers to infectious inflammation of the alveoli, distal airways, and lung interstitium, which can be caused by bacteria, viruses, and other pathogens. Respiratory infections, reported by the Global Burden of Disease (GBD), are the second leading cause of death (Roth et al., 2017), and the incidence of pneumonia per 1,000 people per year is approximately 1.5–14.0 (Millett et al., 2013). Children younger than 5 years and adults older than 65 years are susceptible to pneumonia, and males are more vulnerable to get pneumonia compared with females (Marras et al., 2000; Carratala et al., 2005). The morbidity of pneumonia in children under 5 years is 29% (Morris et al., 2003), and the number of children who died of pneumonia account for almost 30% of the deaths (Rudan et al., 2007). More seriously, a new type of coronavirus is breaking out all over the world and causes severe pneumonia and even death (Yang et al., 2020).

As a common respiratory infection, pneumonia has an excessively high morbidity and mortality rate. It has been a huge burden to the medical system and seriously threatened the quality of human life (Walker et al., 2013). At present, the main treatment of pneumonia is antibacterial, antiviral, antipyretic, and antihistamine drugs. However, due to drug resistance, the clinical application of these drugs is limited (Cornfield, 2017). In recent years, because of fewer side effects and a significant therapeutic effect, traditional Chinese medicine (TCM) has been widely used in enhancing human immunity and in preventing and treating infectious and chronic diseases (Chao et al., 2017; Ma et al., 2019). Since the outbreak of the novel coronavirus in Wuhan, TCM has been actively applied in the prevention and treatment of COVID-19, and remarkable effects have been achieved (Luo et al., 2020; Ren et al., 2020).

Huang-Lian-Jie-Du decoction (HLJDD), derived from the “Elbow Reserve Emergency Prescription,” is composed of Huanglian (HL), Huangbo (HB), Huangqin (HQ), and Zhizi (ZZ). As a classic Chinese medicine formula for clearing away heat and detoxification, HLJDD is widely utilized in the treatment of inflammation-related diseases (Wang et al., 2015), such as pneumonia (Zhao and Wang, 2011) and hepatitis (Wei et al., 2016). Wang and Xu (2000) found that HLJDD can significantly reduce the levels of tumor necrosis factor alpha (TNF-α) and other inflammatory cytokines. Fang et al. (2004) found that HLJDD suppresses inflammation by reducing the level of inflammatory mediators such as PGE2. Research by Xu et al. (2019) reported that HLJDD can reduce the lipopolysaccharide-induced inflammation through the glycerophospholipid metabolism pathway. Furthermore, during the fight against COVID-19, HLJDD also played important roles (Guo and Sun, 2020; Wang et al., 2020). However, our understanding of the common prescription of HLJDD for treating pneumonia is still not clear.

**FIGURE 1:**
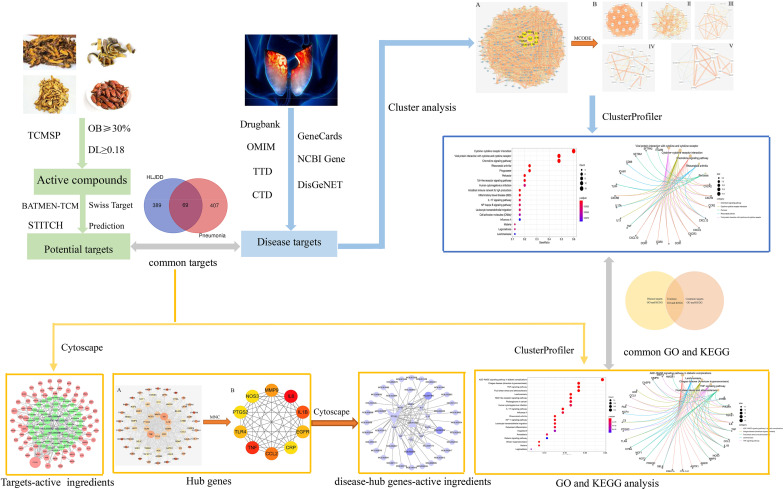
Flowchart of exploring the mechanism of Huang-Lian-Jie-Du decoction (HLJDD) in treating pneumonia. The active ingredients of HLJDD and their potential targets were predicted from different databases. The relevant targets of pneumonia were collected from seven different databases. The intersections of the active ingredient targets and the disease targets were regarded as common targets. The pivotal active ingredients were obtained through a common targets–active ingredients network analysis. The overlapping pathways were obtained through pathway enrichment analysis of the disease targets and common targets. We used the Cytohubba plug-in to select hub genes in the common targets. Finally, the pivotal active ingredients, overlapping pathways, and the hub genes were analyzed to explore the mechanisms of HLJDD in treating pneumonia. The *one-way arrow* indicates the relationship of progressive and the *two-way arrow* indicates the relationship of intersections.

In order to decode the mechanism of HLJDD in the treatment of pneumonia, we explored the targets and pathways of HLJDD in treating pneumonia through network pharmacology. The flowchart of our analysis is shown in Figure 1.

## Materials and Methods

### Active Ingredients in HLJDD

In clinical treatment, drugs are usually administered orally. Oral bioavailability (OB) and drug likeness (DL) are the main variables that affect drug absorption across the gastrointestinal mucosa. Therefore, by using “Huanglian,” “Huangbo,” “Huangqin,” and “Zhizi” as keywords, we screened the active ingredients of HLJDD in the Traditional Chinese Medicine Systems Pharmacology (Ru et al., 2014) (TCMSP^1^, updated on May 31, 2014) with the criteria OB ≥ 30% and DL ≥ 0.18 (Feng et al., 2018). The structural information, molecular structure, “PubChem CID,” and “Canonical SMILES” of the active ingredients were obtained from PubChem^2^ (Kim et al., 2019).

### Targets Related to Active Ingredients

Targets of the active ingredients in HLJDD were obtained from the TCMSP database. Since the targets provided by TCMSP might be incomplete, by using the structure information of the active ingredients obtained from PubChem, we also searched for the targets on three similarity-based target prediction web servers, namely, BATMEN-TCM (Liu et al., 2016), Swiss Target Prediction (Gfeller et al., 2014), and STITCH (Kuhn et al., 2008) to predict the targets of the active ingredients in HLJDD (Wu et al., 2018). BATMEN-TCM^3^ is an online bioinformatics analysis tool for studying the molecular mechanism of TCM (Liu et al., 2016). The parameters of BATMEN-TCM are set as score ≥ 20 and adjusted *p* ≤ 0.05. The Swiss Target Prediction^4^ is an online tool aiming to predict molecular targets (Gfeller et al., 2014). In the Swiss Target Prediction, the species is restricted to “Homo sapiens” and the probability is set as no less than 0.6. STITCH^5^ is a database of known and predicted interactions between chemical molecules and proteins (Kuhn et al., 2008). In the present work, the species “*Homo sapiens*” and a confidence score ≥ 0.6 were set as the parameters of STITCH. Finally, all the obtained genes were standardized by screening in UniProt^6^ (UniProt Consortium, 2015).

### Known Targets of Pneumonia

Targets relevant to pneumonia were collected from the DrugBank database^7^ (Wishart et al., 2006) (updated on July 2, 2020), OMIM database^8^ (Amberger and Hamosh, 2017) (updated on January 8, 2020), TTD database^9^ (Li Y. H. et al., 2018) (updated on January 6, 2020), CTD database^10^ (Davis et al., 2019) (updated on June 26, 2020), GeneCards database^11^ (Stelzer et al., 2016) (updated on 11 March 2020), NCBI Gene database^12^ (Brown et al., 2015) (updated on August 15, 2019), and the DisGeNET database^13^ (Pinero et al., 2015) (updated on June, 2020). Using “pneumonia” as the keyword, we obtained 63, 116, 15, 39, 44, 229, and 191 targets associated with pneumonia from these databases, respectively. These targets were also standardized in UniProt.

### Network Construction

#### PPI Network Construction

String^14^ is a database of known and predicted protein–protein interactions. It currently contains approximately 24.6 million proteins from 5,090 organisms (Szklarczyk et al., 2019). In the present work, potential target interactions were analyzed by using String with the settings of Organism for “*Homo sapiens*” and a confidence score ≥ 0.4.

#### Cluster Analysis

Cluster analysis aims to screen out the same or similar nodes and protein complexes from complex protein–protein interaction (PPI) networks (Anitha et al., 2016). The MCODE plug-in in Cytoscape (version 3.6.1) was used for the cluster analysis of PPI networks (Deng et al., 2019). A node score cutoff = 0.2, *k* core = 2, maximum depth = 100, and a degree cutoff = 2 were set as the filter conditions.

#### Network Visualization and Identification of Hub Genes

By taking the intersection between the targets of HLJDD and pneumonia, their common targets were obtained. A disease targets PPI network, common targets PPI network, common targets–active ingredients network, and a disease hub genes–active ingredients network were constructed and visualized by using Cytoscape^15^ (version 3.6.1) (Shannon et al., 2003). The Cytohubba plug-in was performed for the identification of hub genes, and the top 10 genes generated by the maximum neighborhood component (MNC) method were regarded as hub genes (Yang et al., 2019). The network analyzer plug-in was used to perform interactive network topology analysis. For each node in an interactive network, the degree value is an important parameter to evaluate its topological characteristics, which measures the number of connections with other nodes and reflects the importance of a node (Meng et al., 2019).

### GO and KEGG Pathway Enrichment Analysis

Gene Ontology (GO) annotation is used to define and describe the functions of gene products from three aspects, namely, biological processes (BPs), cell components (CCs), and molecular functions (MFs). The Kyoto Encyclopedia of Genes and Genomes (KEGG) is a database that integrates genome, chemistry, and system function information. In order to obtain more accurate GO and KEGG functional enrichment information, the clusterProfiler software package of the R platform was used to perform GO and KEGG functional enrichment analysis, and the screening criterion was adjusted *p*≤0.05 (Yu et al., 2012).

### Active Ingredients and Hub Targets Interaction Analysis

The crystal structures of the hub targets were downloaded from the RCSB PDB database^16^, and the structures of the active ingredients were obtained from the TCMSP database. Before molecular docking, the AutoDock 4.2.6 software was used to remove ligands, structure water molecules, and add polar hydrogen atoms and charges to the protein crystal structures (Li H. Y. et al., 2018). The Discovery Studio software was used to evaluate the interaction between the targets and the active ingredients.

## Results

### Potential Targets of Active Ingredients

With the criteria of OB ≥ 30% and DL ≥ 0.18, a total of 102 active ingredients were identified in HLJDD based on TCMSP, of which 14 were from HL, 37 from HB, 36 from HQ, and 15 were from ZZ. Detailed information of these active ingredients is listed in Supplementary Table 1. The targets of the 102 active ingredients were predicted by the above-mentioned target prediction web servers (BATMEN-TCM, Swiss Target Prediction, and STITCH). After removing redundant targets, 458 potential targets of the 102 identified active ingredients were obtained, which are provided in Supplementary Table 2.

### Common Targets–Active Ingredients Network

**FIGURE 2:**
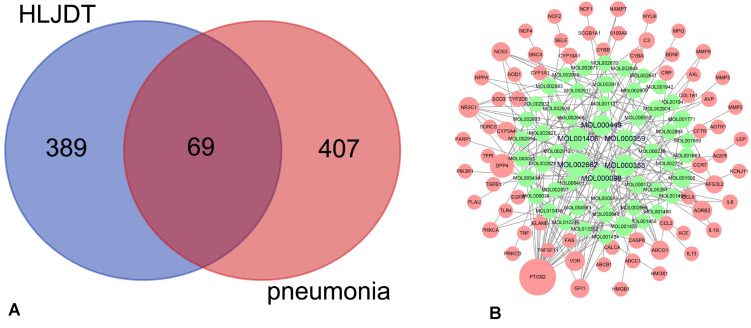
Common targets and common targets–active ingredients network. **(A)** Common targets of Huang-Lian-Jie-Du decoction (HLJDD) and pneumonia. **(B)** Common targets–active ingredients network. *Red nodes* represent the common targets of pneumonia and HLJDD; *green nodes* represent the active ingredients related to the common targets. The six nodes with *bold black fonts in the middle* are the compounds with degree ≥ 7 in the network.

The occurrence and development of pneumonia is a complex process involving the regulation of multiple genes and proteins (Quinton et al., 2018). By screening DrugBank, OMIM, TTD, CTD, GeneCards, NCBI Gene, and DisGeNET, 476 pneumonia-related targets were obtained (Supplementary Table 3). By comparing the targets of HLJDD and pneumonia, we found that pneumonia shares 69 targets with those of the active ingredients (Figure 2A), and the 69 targets were correlated with 54 active ingredients (Supplementary Table 4). The common targets–active ingredients network is shown in Figure 2B. Based on the degree value, we identified six pivotal active ingredients with degree ≥ 7, namely, quercetin (MOL000098), rutaecarpine (MOL002662), sitosterol (MOL000359), beta-sitosterol (MOL000358), crocetin (MOL001406), and stigmasterol (MOL000449).

### PPI Network and Cluster Analysis of Disease Targets

**FIGURE 3:**
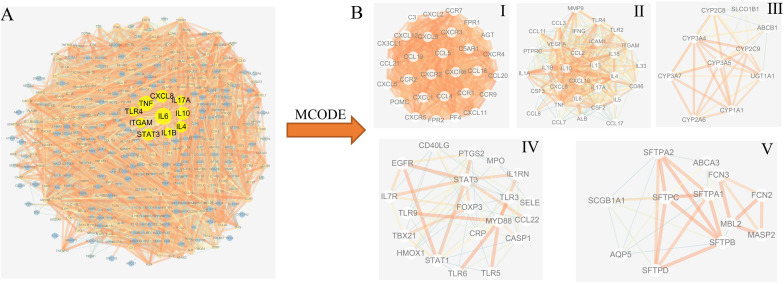
Protein–protein interaction (PPI) network and cluster analysis of the disease targets. **(A)** PPI network of pneumonia targets. The *yellow nodes in the middle* represent the 10 genes with the largest degree values. **(B)** Top five clustering graphs from the PPI network of pneumonia targets.

**FIGURE 4:**
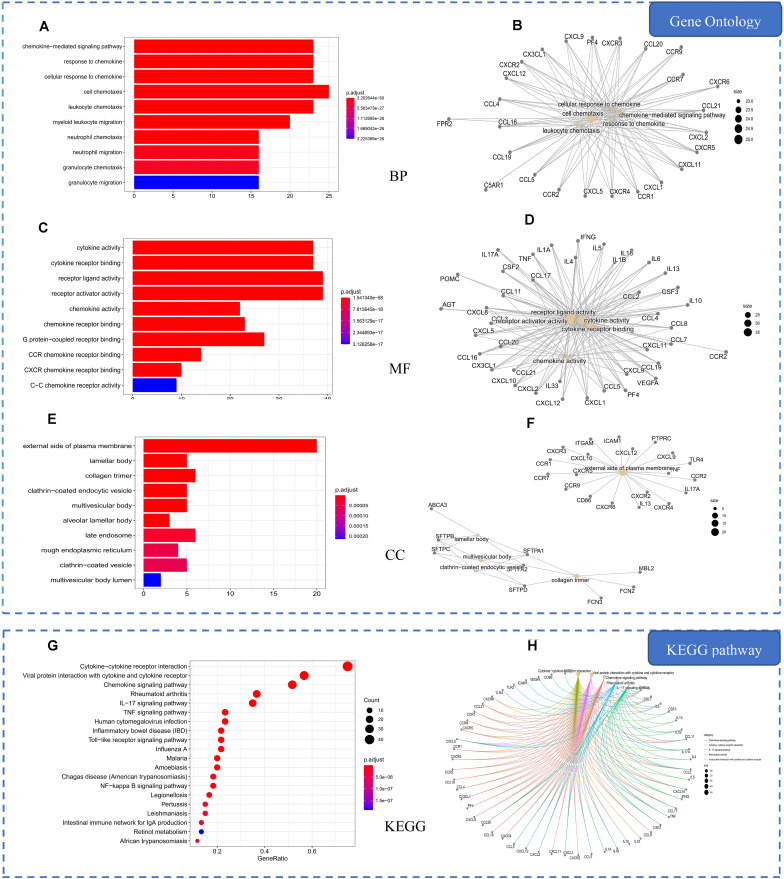
Gene Ontology (GO) and Kyoto Encyclopedia of Genes and Genomes (KEGG) analysis of pneumonia-related genes. **(A)** Top 10 significantly enriched terms in biological processes (BPs). **(B)** Sub-network showing the top five BP terms and related genes. **(C)** Top 10 significantly enriched terms in molecular functions (MFs). **(D)** Sub-network showing the top five MF terms and related genes. **(E)** Top 10 significantly enriched terms in cellular components (CCs). **(F)** Sub-network showing the top five CC terms and related genes. **(G)** The 20 pathways with the lowest adjusted *p* values. The *X*-axis is the GeneRatio of the term and the *Y*-axis is the name of the terms. The *darker the color*, the smaller the adjusted *p* value. The *larger the circle*, the greater the number of the target genes in the term. **(H)** Sub-network showing the top five KEGG pathways and related genes.

Protein–protein interaction network analysis on the pneumonia targets was performed by using the String database online service platform, and the results are shown in Figure 3A. The yellow nodes in the middle represent the 10 genes (i.e., *TLR4*, *TNF*, *CXCLB*, *IL1B*, *IL17A*, *IL10*, *IL4*, *IL6*, *STAT3*, and *ITGAM*) with the largest degree values, which play important roles in the genesis and progression of pneumonia. The MCODE plug-in in Cytoscape was then used to cluster the PPI network of pneumonia targets. Accordingly, 16 clusters were obtained (Supplementary Table 5). According to their scores, we selected the top five clusters (Figure 3B and Table 1) and performed GO functional enrichment and KEGG pathway analysis on the targets covered by the five clusters. Finally, 1,475 BPs, 99 MFs, 46 CCs, and 91 KEGG pathways were obtained (*p* ≤ 0.05). The top 10 significant terms in BPs, MFs, and CCs and the top 20 significant KEGG pathways are shown in Figures 4A,B, respectively.

**TABLE 1:** Cluster information of the pneumonia protein–protein interaction (PPI) network.

Cluster	Score	Nodes	Edges	Gene symbol
1	29	29	406	*CXCR2*, *CXCL1*, *POMC*, *CXCL11*, *CXCL2*, *CXCL12*, *FPR1*, *CCL5*, *CXCR4*, *CCL19*, *CCL20*, *C3*, *CXCL9*, *AGT*, *PF4*, *FPR2*, *CX3CL1*, *CCL4*, *C5AR1*, *CCL16*, *CCR2*, *CXCR3*, *CCR1*, *CXCL5*, *CXCR5*, *CCR9*, *CCR7*, *CCL21*, and *CXCR6*
2	24.867	31	373	*CXCL8*, *CCL8*, *IL33*, *VEGFA*, *IL10*, *CCL7*, *IL6*, *ALB*, *CSF3*, *IL13*, *IL1B*, *CCL11*, *IL18*, *PTPRC*, *IL17A*, *ICAM1*, *TLR4*, *IL4*, *CCL2*, *ITGAM*, *IL1A*, *CD86*, *IL5*, *TLR2*, *CXCL10*, *IFNG*, *CCL17*, *TNF*, *CSF2*, *MMP9*, and *CCL3*
3	8.444	10	38	*CYP2A6*, *CYP3A5*, *CYP3A7*, *CYP3A4*, *ABCB1*, *UGT1A1*, *SLCO1B1*, *CYP2C9*, *CYP2C8*, and *CYP1A1*
4	6.526	20	62	*TLR6*, *FOXP3*, *MPO*, *STAT3*, *CD40LG*, *IL1RN*, *IL7R*, *MYD88*, *EGFR*, *CCL22*, *HMOX1*, *TLR5*, *PTGS2*, *CASP1*, *TLR3*, *CRP*, *TBX21*, *TLR9*, *STAT1*, and *SELE*
5	5.636	12	31	*MASP2*, *MBL2*, *SFTPD*, *FCN3*, *SFTPC*, *SFTPB*, *SFTPA1*, *ABCA3*, *SCGB1A1*, *SFTPA2*, *AQP5*, and *FCN2*

GO enrichment analysis showed that the biological processes of pneumonia are mainly related to chemokines and cell migration, such as response to chemokine, cellular response to chemokine, leukocyte migration, and neutrophil migration. The molecular functions of pneumonia would be related to cytokine activity, cytokine receptor binding, and chemokine receptor binding. The cellular components associated with pneumonia could be activated in lamellar body, external side of the plasma membrane, and rough endoplasmic reticulum (Figure 4A). The KEGG pathway analysis demonstrated that the main pathways associated with pneumonia were cytokine–cytokine receptor interaction, chemokine signaling pathway, and viral protein interaction with cytokine and cytokine receptor (Figure 4B).

### Common Targets Enrichment Analysis

**FIGURE 5:**
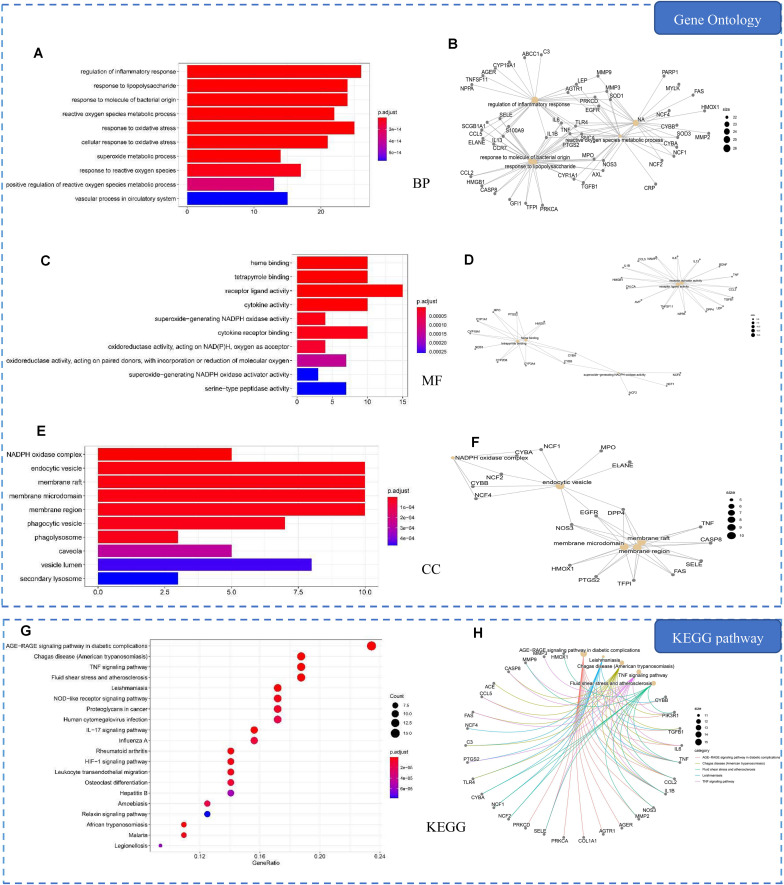
Gene Ontology (GO) and Kyoto Encyclopedia of Genes and Genomes (KEGG) analysis of common targets. **(A)** Top 10 significantly enriched terms in biological processes (BPs). **(B)** Sub-network showing the top five BP terms and related genes. **(C)** Top 10 significantly enriched terms in molecular functions (MFs). **(D)** Sub-network showing the top five MF terms and related genes. **(E)** Top 10 significantly enriched terms in cellular components (CCs). **(F)** Sub-network showing the top five CC terms and related genes. **(G)** The 20 pathways with the lowest adjusted *p* values. The *X*-axis is the GeneRatio of the term and the *Y*-axis is the name of the terms. The *darker the color*, the smaller the adjusted *p* value. The *larger the circle*, the greater the number of target genes in the term. **(H)** Sub-network showing the top five KEGG pathways and related genes.

In order to explore the mechanism of HLJDD in the treatment of pneumonia, we used the ClusterProfiler software package of the R platform to perform KEGG and GO functional enrichment analysis on the 69 common targets. Accordingly, we obtained 1,730 BPs, 110 MFs, 53 CCs, and 88 KEGGs (*p* ≤ 0.05). The top 10 significant terms in BPs, MFs, and CCs and the 20 significant KEGG pathways are shown in Figure 5.

GO enrichment analysis demonstrated that the common targets are mainly related to inflammatory response, bacteria, and oxidative stress, such as response to oxidative stress, response to molecule of bacterial origin, and regulation of inflammatory response (Figure 5A). The main molecular functions of common targets are heme binding, tetrapyrrole binding, and receptor ligand activity. The cellular component would be mainly activated in the endocytic vesicle, membrane raft, and membrane microdomain. The most significant pathways of common targets are the AGE–RAGE signaling pathway in diabetic complications, Chagas disease (American trypanosomiasis), and the TNF signaling pathway (Figure 5B).

### Identification of Hub Genes

**FIGURE 6:**
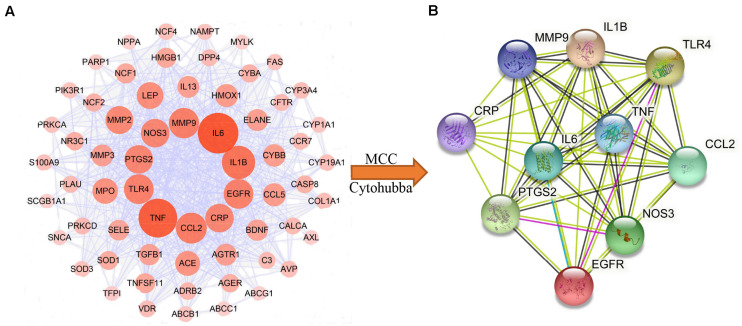
Identification of hub genes of Huang-Lian-Jie-Du decoction (HLJDD) for pneumonia. **(A)** Sixty-nine common targets protein–protein interaction (PPI) network. This network has 69 nodes and 626 edges. **(B)** PPI network of the hub genes.

In order to obtain the hub genes of HLJDD in treating pneumonia, we firstly imported the 69 common targets into the String database and constructed their PPI network. Subsequently, the obtained PPI network was imported into the Cytoscape platform, and the Cytohubba plug-in was used for the identification of hub genes. The top 10 nodes in the network generated by the MNC method were regarded as hub genes (Figure 6A), i.e., *IL1B*, *IL6*, *CCL2*, *MMP9*, *PTGS2*, *TNF*, *CRP*, *EGFR*, *TLR4*, and *NOS3*. *TLR4*, *TNF*, *IL1B*, and *IL6* are also important pneumonia targets. The PPI network of the 10 hub genes has 10 nodes and 44 edges, with an average node degree of 8.8 and *p* value of 7.89e-12 (Figure 6B).

### Selecting and Analyzing Critical GO and KEGG of HLJDD on Pneumonia

After comparing and analyzing the KEGG and GO functions of the disease targets and common targets of HLJDD and pneumonia, we found 971 overlapping BPs, 47 overlapping MFs, 27 overlapping CCs, and 58 overlapping KEGG pathways (Supplementary Table 6).

The hub genes selected by the MNC method may play a more important role in the treatment of pneumonia *via* HLJDD. Therefore, we selected the GO and KEGG that contain more than five hub genes for further analysis. Accordingly, we obtained six critical biological processes, three critical molecular functions, two critical cellular components, and five critical KEGG pathways, which are highlighted in red in Supplementary Table 6. Although the number of hub genes enriched in the MAPK signaling pathway and NF-kappa B signaling pathway were less than five, they are important pneumonia-related pathways (Zhang et al., 2019a). Therefore, they were also screened in the following analysis.

The critical molecular functions were mainly related to receptor ligand activity (GO:0048018), cytokine activity (GO:0005125), and cytokine receptor binding (GO:0005126). The critical cellular components were membrane raft (GO:0045121) and membrane microdomain (GO:0098857). The critical BPs and KEGG pathways were mainly enriched in the categories of bacterial stimulation, immune response, signal transduction, and inflammatory response. In terms of bacterial stimulation, the categories of BPs were response to lipopolysaccharide (GO:0032496), response to molecule of bacterial origin (GO:0002237), cellular response to lipopolysaccharide (GO:0071222), cellular response to molecule of bacterial origin (GO:0071219), and cellular response to biotic stimulus (GO:0071216). The categories of KEGG pathways were Chagas disease (hsa05142) and *Yersinia* infection (hsa05135). In terms of immune response, the categories of KEGG pathways were IL-17 signaling pathway (hsa04657) and nucleotide-binding oligomerization domain (NOD)-like receptor signaling pathway (hsa04621). In terms of inflammatory response, the category of BPs was regulation of inflammatory response (GO:0050727), and the category of KEGG pathways was TNF signaling pathway (hsa04668). In terms of signal transduction, the categories of KEGG pathways were MAPK signaling pathway (hsa04010) and NF-kappa B signaling pathway (hsa04064).

### Disease Hub Genes–Active Ingredients–Herbs Network

**FIGURE 7:**
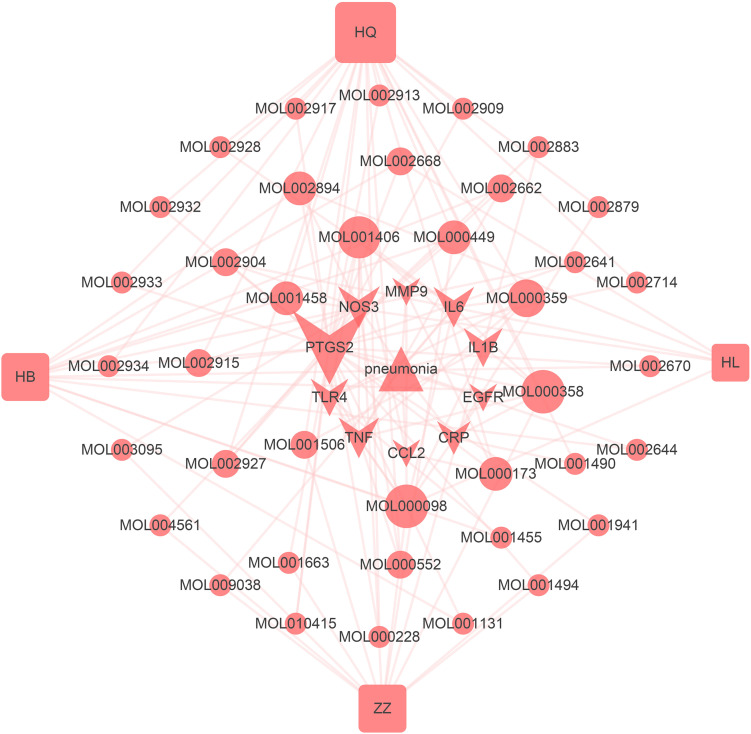
Disease hub gene–active ingredient–herb network. *Triangle nodes* represent disease, *arrow-like nodes* represent hub genes, *circle nodes* represent the active ingredients related to the hub genes, and the *square nodes* represent herbs. The size of each node was set according to their degree value.

Based on the active ingredients related to the hub genes, the disease targets–active ingredients–herbs network was constructed (Figure 7). This network has 54 nodes and 126 edges (4 herbs, 39 active ingredients, 10 hub genes, and 1 disease). It can be seen that HQ has the most active ingredients that act on hub genes. This is because HQ is good at clearing the heat of the lungs (Wang, 2015). Quercetin (MOL000098), rutaecarpine (MOL002662), sitosterol (MOL000359), beta-sitosterol (MOL000358), crocetin (MOL001406), and stigmasterol (MOL000449) are pivotal ingredients that were identified by the common targets–active ingredients network, which might be the material basis for HLJDD in treating pneumonia.

### Validation by Molecular Docking

**FIGURE 8:**
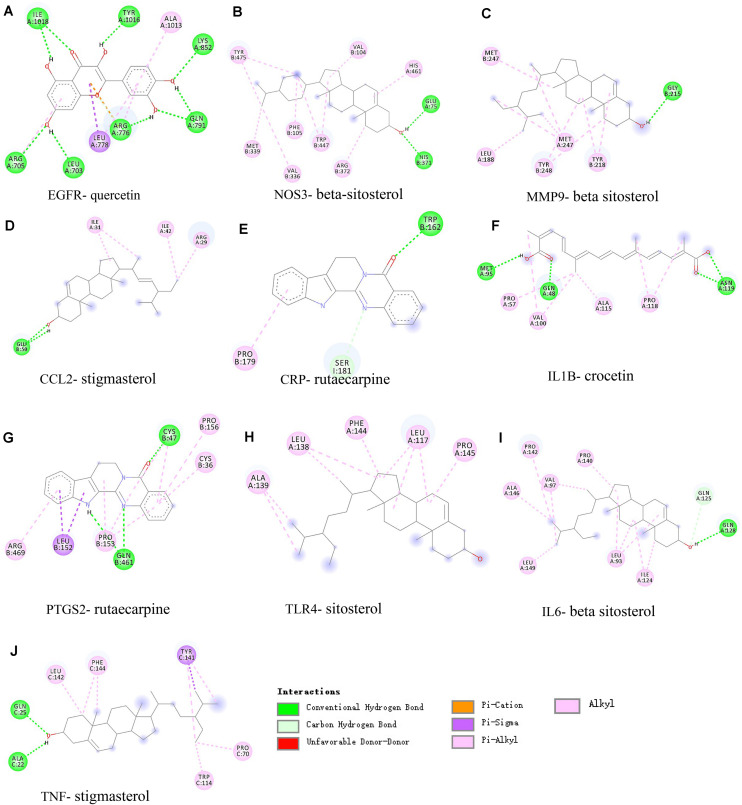
Molecular docking of active ingredients and hub targets. **(A)** *EGFR*, **(B)** *NOS3*, **(C)** *MMP9*, **(D)** *CCL2*, **(E)** *CRP*, **(F)** *IL1B*, **(G)** *PTGS2*, **(H)** *TLR4*, **(I)** *IL6*, and **(J)** *TNF*. *Different colors* represent the different interactions between the active ingredients and the proteins.

The 10 hub targets and six pivotal active ingredients were used as receptors and ligands, respectively. The docking results are listed in Supplementary Table 7. The stability of the binding of the receptors and ligands depends on the binding energy. The lower the binding energy, the more stable the binding conformation of the receptor and the ligand. The binding energy −5.0 kcal/mol is set as the threshold to determine whether the binding of the receptors and ligands is good or not (Li et al., 2020). Therefore, we screened out the ligand which binds the best (with the lowest energy) to the receptor based on the binding energy. The screening results are shown in Table 2, and the interaction between the receptors and the ligands are shown in Figure 8.

**TABLE 2:** Screening docking results between ligands and receptors.

Hub targets (PDB ID)	Active ingredients	Binding energy (kcal/mol)
IL1B (31BI)	Crocetin	−7.6
IL6 (1IL6)	Sitosterol	−7.98
NOS3 (3NOS)	Sitosterol	−8.31
PTGS2 (5F19)	Rutaecarpine	−8.34
TLR4 (2Z62)	Beta-sitosterol	−7.79
TNF (2ZJC)	Stigmasterol	−7.72
CCL2 (1DOM)	Stigmasterol	−7.55
CRP (1LJ7)	Rutaecarpine	−6.52
EGFR (2GS2)	Quercetin	−8.81
MMP9 (4H3X)	Sitosterol	−10.24

As indicated in Table 2, all active ingredients can bind well to the hub targets. The docking of the hub targets and pivotal active ingredients was mainly through the following seven interaction forms, including conventional hydrogen bond, unfavorable donor–donor, carbon–hydrogen bond, pi–sigma, pi–cation, pi–alkyl, and alkyl (Figure 8). Beta-sitosterol has the best binding to MMP9 through 10 interactions, mainly including pi–sigma and conventional hydrogen bond. These results indicate that the active ingredients have a better binding performance to protein targets.

## Discussion

Pneumonia was caused either by bacteria or viruses, called bacterial pneumonia and viral pneumonia, respectively. As the eighth leading cause of death in the world, pneumonia still has a high mortality (Heron, 2016). As a traditional Chinese medicine, HLJDD has been widely used to treat pneumonia for thousands of years. However, its mechanisms of clinical effects remain unclear. Therefore, we explored the underlying mechanisms of the therapeutic effect of HLJDD in the treatment of pneumonia by means of network pharmacology.

By constructing the PPI network for the common targets of HLJDD and pneumonia, *IL1B*, *IL6*, *CCL2*, *MMP9*, *PTGS2*, *TNF*, *CRP*, *EGFR*, *TLR4*, and *NOS3* were found to be the hub genes of HLJDD in treating pneumonia. These genes are mainly related to host immunity, oxidative stress, bacteria, and microorganisms and are covered by the TNF signaling pathway (*IL6*, *TNF*, *CCL2*, *MMP9*, *CRP*, *IL1B*, *PTGS2*, and *EGFR*), IL-17 signaling pathway (*IL6*, *TNF*, *CCL2*, *MMP9*, *IL1B*, *PTGS2*, and *EGFR*), and Chagas disease pathway (*IL6*, *TNF*, *TLR4*, *CCL2*, *CRP*, *IL1B*, and *EGFR*). We noticed that only four of the hub genes were overlapped with the 10 important genes of pneumonia. This might explain why HLJDD is only effective for special kinds of pneumonia rather than for all kinds of pneumonia.

According to the analysis of the hub genes, pivotal active ingredients, and main significant KEGG pathways, the potential mechanisms of HLJDD in the treatment of pneumonia might be attributed to the following aspects.

### Regulate Host Immune Inflammatory Response and Oxidative Stress

IL-6 is an important marker of inflammatory response. It can lead to the upregulation of Th17/Treg balance, which is pathologically involved in the development of chronic inflammatory diseases (Kimura and Kishimoto, 2010; Tanaka et al., 2018). Downregulation of the inflammatory factor IL-6 can treat lipopolysaccharide (LPS)-induced pneumonia effectively. By comparing patients with chronic obstructive pulmonary disease (COPD) with a normal group, Gao et al. found that *IL1B* and *CCL2* were the most upregulated genes in COPD small airway epithelial cells and IL1B is an airway inflammatory molecule in COPD (Yi et al., 2018). Xue et al. (2019) found that the serum level of CCL2 in patients with idiopathic interstitial pneumonia was significantly higher than that of the normal control group, and it can affect macrophages, T lymphocytes, and the production of antibodies. TLR4 was found to be the direct target of miR-370-3p, and its signaling can promote the occurrence of acute or chronic pneumonia and inhibit vascular inflammation and oxidative stress by downregulating the expression of the TLR4 protein (Tian et al., 2018; Zhang et al., 2019b). In addition, by performing the KEGG analysis, we found that the synthesis of inflammatory mediators and inflammatory cytokines mediated by the TNF signaling pathway and the pro-inflammatory cytokines mediated by the NOD-like receptor signaling pathway were all related to the inflammation formation of the lungs. Leukocyte recruitment mediated by the TNF signaling pathway and autoimmune pathology mediated by the IL-17 signaling pathway were related to host immune response. The NOD-like receptor signaling pathway is related to innate immune response, which can regulate the maturation of pro-inflammatory cytokines, i.e., IL1B, IL6, and TNF, and trigger cell apoptosis.

The pivotal active ingredients related to host immunity and inflammation were also obtained. Rutaecarpine (MOL002662) can inhibit the production of pro-inflammatory factors IL6 and IL1B, thereby reducing the inflammatory response and oxidative stress caused by cerebral ischemia–reperfusion (CI/R) and increasing the levels of anti-inflammatory factors and superoxide dismutase (Han et al., 2019). Quercetin (MOL000098) and crocetin (MOL001406) can enhance immunity, inhibit cell apoptosis, and reduce the levels of the inflammatory factors IL6 and TNF-α, thereby alleviating the inflammatory damage of lung fibroblasts (Yang et al., 2012; Wang et al., 2019). Aaron et al. found that stigmasterol (MOL000449) can control oxidative stress and retain the antioxidant capacity of the lung tissue (Antwi et al., 2017).

### Anti-Parasitic Pneumonia Caused by Bacteria and Microorganisms

Bacteria can break the alveolar wall (causing leakage of protein fluid into the alveoli), block pulmonary blood vessels, activate platelet-activating factor receptors, change the permeability of endothelial cells, and mediate the activity of Src family kinases, thereby making the lungs susceptible to parasitic infections and forming parasitic pneumonia (Cheepsattayakorn and Cheepsattayakorn, 2014). IL1B is a pro-inflammatory cytokine, and its production is triggered by the activation of pattern recognition receptors by microbial products. IL1B can induce prostaglandin synthesis, neutrophil influx and activation, T cell activation, and cytokine production (Nakahara et al., 2003; Dinarello, 2009). Yang et al. (2014) found that activated NOS3 can enhance the ability of lung macrophages to eliminate bacteria, thereby increasing the host’s resistance to bacterial pneumonia. C-reactive protein (CRP) can be used as one of the biomarkers to identify bacterial pneumonia and viral pneumonia (Bhuiyan et al., 2019). Neutrophil recruitment mediated by the IL-17 signaling pathway is closely related to bacterial infection. *Trypanosoma cruzi* is an intracellular protozoan parasite that causes Chagas disease. It can also invade lung cells and affect the host’s innate immunity and interference T lymphocytes. The TLR4–TRAF6–AP1–TNFα pathway in Chagas disease and *Yersinia* infection can indirectly inhibit the growth of parasites and inhibit the response of pro-inflammatory cytokines by regulating TNFα, IL6, and IL1B. Beta-sitosterol (MOL000358) and sitosterol (MOL000359) have antimicrobial and immunomodulatory effects. Its derivative 2-naphthoyl Sit ester can inhibit the overexpression of TLR4 induced by LPS, thereby inhibiting inflammation and regulating antioxidants (Yin et al., 2018).

### Alleviate the Clinical Symptoms of Pneumonia

Ge et al. (2019) found that the activation of the MAPK pathway by inhibiting PTGS2 can reduce the inflammatory response. LPS stimulates the synthesis of IL1B and promotes its cytoplasmic storage (Andrei et al., 2004). IL1B induces the synthesis of prostaglandins (PGs) that affect the pneumonia response, and PTGS2 acts as a key rate-limiting enzyme in the synthesis of PGs. Therefore, the effects of inflammation could be reduced by regulating IL1B and PTGS2 (COX-2) that control the synthesis of PGs (Van Damme et al., 1985; Li et al., 2019). Zhang et al. (2019a) reported that regulating the NF-κB/MAPK signaling pathway can reduce pulmonary inflammation in rats. Carlsen et al. (2015) found that quercetin (MOL000098) can restore the high expression of PTGS2 caused by inflammation to normal levels. It has been shown that the expression of the inflammatory mediator PTGS2 was inhibited by crocetin (MOL001406) in an inflammation model. Crocetin exerted an anti-inflammatory effect and improved inflammatory symptoms by suppressing the activation of the MAPK signaling pathway (Hemshekhar et al., 2012).

### Repairing Damaged Cells and Inhibiting Cell Migration

Matrix metalloproteinase-9 plays an important role in the local proteolysis of the extracellular matrix and leukocyte migration (Tschesche et al., 1992). When epithelial cells are damaged, the body can repair the damaged epithelial cells by inhibiting the expression of MMP9 (Widgerow, 2011). Inhibition of MMP3 and MMP9 expressions precludes the migration of vascular smooth muscle cells caused by pneumonia infection (Ma et al., 2015). Transactivation of epidermal growth factor receptor (EGFR) can protect alveolar epithelial cells from TNF-induced cell damage. Based on KEGG analysis, we also found that the TNF and IL-17 signaling pathways promote extracellular matrix and tissue remodeling by regulating MMP3 and MMP9. Quercetin (MOL000098) can attenuate cell migration and invasion by inhibiting the expression level of MMP9 through the NF-kappa B signaling pathway (Lu et al., 2018; Cheng et al., 2019).

## Conclusion

We obtained the pivotal active ingredients of HLJDD in treating pneumonia, namely, quercetin, rutaecarpine, sitosterol, beta-sitosterol, crocetin, and stigmasterol. By constructing a compound–target–disease network, 10 hub genes were screened out, i.e., *IL1B*, *IL6*, *CCL2*, *MMP9*, *PTGS2*, *TNF*, *CRP*, *EGFR*, *TLR4*, and *NOS3*. The significant biological processes, molecular functions, cellular components, and KEGG pathways were obtained by performing GO and KEGG pathway analysis. The pivotal active ingredients, hub genes, and enrichment analysis results indicate that HLJDD may have a greater effect on bacterial pneumonia than on viral pneumonia, which can explain why HLJDD does not appear frequently in the process of using Chinese medicine to prevent and treat COVID-19 (Wang et al., 2020). The therapeutic effect of HLJDD in treating pneumonia is mainly by regulating the host’s immune inflammatory response and oxidative stress, antibacterial microorganisms, relieving the clinical symptoms of pneumonia, repairing damaged cells, and inhibiting cell migration.

## Data Availability Statement

The original contributions presented in the study are included in the article/Supplementary Material, further inquiries can be directed to the corresponding author/s.

## Author Contributions

WC and XL conceived and designed the experiments. XL and QT performed the experiments. XL, HT, and WC wrote the manuscript. All authors read and approved the final manuscript.

## Conflict of Interest

The authors declare that the research was conducted in the absence of any commercial or financial relationships that could be construed as a potential conflict of interest.

## Footnotes

1 https://tcmspw.com/

2 https://pubchem.ncbi.nlm.nih.gov/

3 http://bionet.nb.org/batman-tcm/

4 http://www.swisstargetprediction.ch

5 http://stitch.embl.de/

6 http://www.uniprot.org/

7 https://www.drugbank.ca

8 http://omim.org/

9 http://bidd.nus.edu.sg/group/cjttd/

10 http://ctdbase.org/

11 https://www.genecards.org/

12 https://www.ncbi.nlm.nih.gov

13 https://www.disgenet.org

14 https://string-db.org/

15 http://www.cytoscape.org

16 http://www.rcsb.org/

## Abbreviations

HLJDD: Huang-Lian-Jie-Du decoction
IL1B: interleukin-1 beta
IL6: interleukin-6
CCL2: C–C motif chemokine 2
MMP9: matrix metalloproteinase-9
PTGS2: prostaglandin G/H synthase 2
TNF: tumor necrosis factor
CRP: C-reactive protein
EGFR: epidermal growth factor receptor
TLR4: toll-like receptor 4
NOS3, nitric oxide synthase: endothelial
Treg: regulatory T cells
Th17: T helper cell 17
CI/R: cerebral ischemia–reperfusion.
