# Supplementary material for: Decoding the Mechanism of Huanglian Jiedu Decoction in Treating Pneumonia Based on Network Pharmacology and Molecular Docking
Source: Front Cell Dev Biol. 2021 Feb 18;9:638366. doi: 10.3389/fcell.2021.638366 (PMC7930397; doi:10.3389/fcell.2021.638366)
Supplement: Supplementary file 1 [file Data_Sheet_1.ZIP › Table S7.docx]

Table S7 Molecular docking results of hub targets and pivotal active ingredients

| **Hub targets** | **Quercetin**  **(Kcal/mol)** | **Rutaecarpine**  **(Kcal/mol)** | **Sitosterol**  **(Kcal/mol)** | **Beta sitosterol**  **(Kcal/mol)** | **Crocetin**  **(Kcal/mol)** | **Stigmasterol**  **(Kcal/mol)** |
| --- | --- | --- | --- | --- | --- | --- |
| **IL1B** | -6.32 | -6.73 | -7.1 | -6.67 | -7.6 | -6.14 |
| **IL6** | -6.44 | -7.69 | -7.98 | -7.9 | -6.53 | -7.93 |
| **NOS3** | -5.93 | -7.37 | -8.31 | -7.88 | -6.02 | -7.37 |
| **PTGS2** | -6.34 | -8.34 | -7.13 | -7.01 | -6.34 | -7.99 |
| **TLR4** | -5.68 | -7.21 | -7.69 | -7.79 | -6.62 | -7.55 |
| **TNF** | -4.96 | -6.5 | -6.78 | -6.81 | -4.36 | -7.72 |
| **CCL2** | -4.78 | -6.37 | -7.07 | -6.6 | -7.0 | -7.55 |
| **CRP** | -3.89 | -6.52 | -5.68 | -6.09 | -5.11 | -6.05 |
| **EGFR** | -8.81 | -8.31 | -7.29 | -7.46 | -6.88 | -7.83 |
| **MMP9** | -6.45 | -8.42 | -10.24 | -9.2 | -7.72 | -9.78 |
